# Models of care for orphaned and separated children and upholding children’s rights: cross-sectional evidence from western Kenya

**DOI:** 10.1186/1472-698X-14-9

**Published:** 2014-04-01

**Authors:** Lonnie Embleton, David Ayuku, Allan Kamanda, Lukoye Atwoli, Samuel Ayaya, Rachel Vreeman, Winstone Nyandiko, Peter Gisore, Julius Koech, Paula Braitstein

**Affiliations:** 1 Department of Medicine, Moi University, College of Health Sciences, Eldoret, Kenya; 2 College of Health Sciences, Department of Behavioral Sciences, Moi University, Eldoret, Kenya; 3 Moi Teaching and Referral Hospital, Eldoret, Kenya; 4 College of Health Sciences, Department of Mental Health, Moi University, Eldoret, Kenya; 5 College of Health Sciences, Department of Child Health and Pediatrics, Moi University, Eldoret, Kenya; 6 Department of Pediatrics, Indiana University, Indianapolis, USA; 7 Academic Model Providing Access to Healthcare, Eldoret, Kenya; 8 Department of Medicine, Indiana University, 1001 West 10th Street, OPW M200 Indianapolis, IN, USA; 9 Dalla Lana School of Public Health, University of Toronto, Toronto, Canada; 10 Regenstrief Institute Inc., Indianapolis, USA

**Keywords:** Orphans, Vulnerable children, Sub-saharan africa, Kenya, Street children, Children’s rights

## Abstract

**Background:**

Sub-Saharan Africa is home to approximately 55 million orphaned children. The growing orphan crisis has overwhelmed many communities and has weakened the ability of extended families to meet traditional care-taking expectations. Other models of care and support have emerged in sub-Saharan Africa to address the growing orphan crisis, yet there is a lack of information on these models available in the literature. We applied a human rights framework using the United Nations Convention on the Rights of the Child to understand what extent children’s basic human rights were being upheld in institutional vs. community- or family-based care settings in Uasin Gishu County, Kenya.

**Methods:**

The Orphaned and Separated Children’s Assessments Related to their Health and Well-Being Project is a 5-year cohort of orphaned children and adolescents aged ≤18 year. This descriptive analysis was restricted to baseline data. Chi-Square test was used to test for associations between categorical /dichotomous variables. Fisher’s exact test was also used if some cells had expected value of less than 5.

**Results:**

Included in this analysis are data from 300 households, 19 Charitable Children’s Institutions (CCIs) and 7 community-based organizations. In total, 2871 children were enrolled and had baseline assessments done: 1390 in CCI’s and 1481 living in households in the community. We identified and described four broad models of care for orphaned and separated children, including: institutional care (sub-classified as ‘Pure CCI’ for those only providing residential care, ‘CCI-Plus’ for those providing both residential care and community-based supports to orphaned children , and ‘CCI-Shelter’ which are rescue, detention, or other short-term residential support), family-based care, community-based care and self-care. Children in institutional care (95%) were significantly (p < 0.0001) more likely to have their basic material needs met in comparison to those in family-based care (17%) and institutions were better able to provide an adequate standard of living.

**Conclusions:**

Each model of care we identified has strengths and weaknesses. The orphan crisis in sub-Saharan Africa requires a diversity of care environments in order to meet the needs of children and uphold their rights. Family-based care plays an essential role; however, households require increased support to adequately care for children.

## Background

Sub-Saharan African communities have been faced with the growing challenge of providing care for orphaned and separated children. The continent accounts for approximately 69% of people currently living with HIV worldwide [1]. The epidemic has left millions of children without one (single orphan) or both parents (double orphan) and many virtually orphaned due to the absence of one or both of their biological parents (separated) [2,3]. Sub-Saharan Africa is home to approximately 55 million orphaned children, 27% orphaned due to AIDS [4,5], accounting for 89% of the world’s AIDS orphans [4,6]. There are approximately 2.6 million orphans in Kenya, 46% orphaned due to HIV/AIDS, representing 13% of children under 18 in the country [5]. The growing orphan crisis has overwhelmed many communities and has weakened the ability of extended families to meet traditional care-taking expectations [7-9].

When one parent dies, many orphans remain with the other parent. Paternal orphans usually stay with their mothers; however, maternal orphans are much less likely to remain with their father [6]. Over 90% of all double orphans and single orphans not living with a surviving parent are cared for in extended families [6,10]. While it is preferred that children be cared for in the community by extended family [6,11-13], in communities where the AIDS epidemic has advanced, there may be fewer available caregivers and an increasing number of overwhelmed and dissolving households. With growing numbers of orphans requiring care and support in combination with high levels of poverty, rapid urbanization, and dissolution of households in sub-Saharan Africa, extended families may not be able to carry out care-taking expectations and responsibilities [7,9,14-18].

In light of the increased burden of orphan care, other models of care and support have emerged in sub-Saharan Africa to address the growing orphan crisis, including institutional care (orphanages) and community-based care [19-22]. Community-based care refers to support programs administered by non-governmental organizations, religious groups, or community-based organizations (CBO’s) that typically enable children to remain in family-based care environments [20,23]. However, there are limited descriptions of these different models in the literature [20,24] or data on their resources, ability to provide basic needs, and protect the rights of orphaned and separated children. UNICEF and others have said that institutional care is not a viable solution and have recommended that countries move toward the de-institutionalization of orphaned children [6,25-27]. In Save the Children’s report ‘A Last Resort: The Growing Concern about Children in Residential Care’ the organization states: *“Save the Children argues that many features of residential care are an abuse of children’s rights…”*[26]; yet there is a lack of empiric evidence to support this assertion.

The United Nations Convention on the Rights of the Child outlines the basic human rights children are entitled to including: the right to survival; to develop to the fullest; to protection from harmful influences, abuse and exploitation; and to participate fully in family, cultural and social life. The Convention protects children's rights by setting standards in health care; education; and legal, civil and social services [28]. Kenya ratified the Convention in 1990, indicating they have committed themselves protecting and ensuring children’s rights and they have agreed to hold themselves accountable for this commitment before the international community [29].

Currently, there are no country-level statistics on orphaned children in different forms of care available in Kenya. This is the first comprehensive paper to report on the models of care for orphaned and separated children in Kenya and therefore provides extremely valuable data and descriptions of these models that are relevant to inform policy surrounding orphan care in Kenya and east Africa. The aim of this paper is to characterize the models of care for children who are orphaned or separated. We applied a human rights framework using 10 indicators from the United Nations Convention on the Rights of the Child [30] to understand what extent children’s basic human rights were being respected in institutional vs. community- or family-based care settings (Table 1). We utilize this framework while describing and characterizing the models of care for orphaned and separated children in Uasin Gishu (UG) County, western Kenya.

**Table 1:** Children’s human rights framework

**Rights of the Child**	**Manifestation**	**Indicators**	**Observations in UG County Kenya**
**Article 7:** The child shall be registered after birth and have the right from birth to a name and nationality, the right to know and be cared for by his or her parents.	• Name and nationality	• Birth Certificate Knowledge of family and interaction/regular contact with family	• Extremely difficult to obtain birth certificates for children in family-based and institutional care.
			• Most children have knowledge of their family in institutional care
**Article 8:** Right to preserve identity, including nationality, name and family relations	• Name and kinship	• Family Connections Programs	• Family connections important component of institutional and community-based care programming
	• Knowledge and memories of personal and family origin		
**Article 14:** Right to freedom of thought, consciences and religion	• Flexibility/space for child’s exploration and expression of different views	• Policy on participation in religious activities	• Compulsory religious education at half of the institutions
			• Many institutions are faith-based organizations
**Article 17:** Right to information	• Ability of the child to access information and knowledge	• Presence of books	• Books rarely available in family-based settings
		• Information and education on HIV prevention	• Both families and institutions provide HIV prevention education
		• Knowledge of parental/family history	• Children in families and institutions have knowledge of family history
**Article 19:** Right to be protected from all forms of physical or mental violence, injury or abuse, neglect or negligent treatment, maltreatment or exploitation	• Protected from any form of maltreatment from caregiver(s)	• The use of corporal punishment to enforce discipline	• Families mainly use corporal punishment as discipline and some institutions; yet it is against the Kenyan constitution
**Article 24:** Right to health	• Accessible healthcare	• Health insurance	• Children in family-based and institutional care are rarely medically insured
**Article 27:** Right to a standard of living adequate for the child’s physical, mental, spiritual, moral and social development	• Adequate protection from the elements	• Type of shelter	• Institutions more likely to provide basic material needs than families
	• Secure dwelling	• Possessing at least one pair of shoes, one blanket, 2 pairs of non-school clothing	
			• Lower standards of living in family-based care in comparison to institutions
	• Basic material needs		
			• Children lack their own mattress, private cabinet, and blankets in family-based care
		• Private cabinet	
**Article 28:** Right to Education	School planning and participation	• School attendance	• Majority of school-aged children attending school in both families and institutions
**Article 31:** Right to rest and leisure, to engage in play and recreational activities	• Flexibility/space for child to play and engage in recreational activities	• Scheduled leisure time	• Toys and games rarely available to children living in family-based care
		• Access to toys, games	• Both institutions and families have space or facilities for sports.
		• Space or facilities for sports	• Lack of scheduled leisure time for children in family-based care
	• Access to equipment		
**Article 32:** Right of the child to be protected from economic exploitation and from performing any work that is likely to be hazardous or to interfere with the child’s education	• Protection from child labour and excessive work	• Household tasks that children assist with	• Children in family-based care assist with many household tasks including firewood and water collection and income generating activities which may interfere with a child’s education as these tasks maybe time consuming.

## Methods

### Study setting

UG County is one of the 47 counties of Kenya, with its headquarters in Eldoret, about 375 kilometers northwest of Kenya’s capital city, Nairobi. In 2010, UG County had approximately 894 179 individuals from 202 291 households, of whom 41.5% are aged 14 years or less [31]. Approximately 51.3% of the population in UG County live below the Kenyan poverty line (1,562 KES pp/month ~ 18.75 USD) [32]. Eldoret has a total population of 289 389 and is currently, the 5th largest city in the country. It is home to Moi University (including Kenya’s 2^nd^ medical school), Moi Teaching and Referral Hospital (MTRH), and the USAID-AMPATH (Academic Model Providing Access to Healthcare) Partnership [33,34]. A long-standing partnership between Moi University, Moi Teaching and Referral Hospital, and a consortium of universities led by Indiana University form the AMPATH Consortium. The program is headquartered in Eldoret, Kenya. Through this consortium, there is a large, multi-disciplinary, and well-established research program. The Principal Investigators of the study are from Indiana University and Moi University, respectively, and both live in Eldoret, the capital of the county of Uasin Gishu. There are numerous orphanages and other organizations serving OVC in the county, and thousands more OVC living in the community. The research team therefore chose to conduct this study in this geographic location because of all of these reasons.

### OSCAR’s health and well-being project

The Orphaned and Separated Children’s Assessments Related to their (OSCAR’s) Health and Well-Being Project is a 5-year longitudinal cohort study evaluating the effects of different care environments on the physical and mental health outcomes of orphaned and separated children aged 18 years of age or less. The study intends to describe these care environments, determine whether they are able to meet basic socioeconomic needs of the resident children, and examine the effect of care environment on resident children’s physical and mental health over time. The study began enrolling participants in June 2010.

### Human subjects protection

This study was approved by the Moi University College of Health Sciences and MTRH Institutional Research and Ethics Committee and the Indiana University Institutional Review Board. Informed consent was provided by the head of household, Director of CCI, and in the case of the street youth, by the District Children’s Officer (DCO). Individual written assent was provided by each child aged 7 years and above. Fingerprints were used for both children and guardians who were unable to sign or write their name.

### Study population

The project follows a cohort of orphaned and separated children from communities within 8 administrative Locations, and includes 300 households, 19 Charitable Children’s Institutions and 100 street-involved children and youth in UG County of western Kenya [35]. The present analysis was restricted to baseline data collected June 2010-April 2013 and excludes data collected concerning street-involved children and youth. In addition, for the specific aim of this paper, the project recruited CBO's providing community-based services to orphaned and vulnerable children in Eldoret town to participate in a one-time survey to describe their contributions to orphan care.

### Eligibility, sampling and recruitment

#### Family-based care environments

The project aimed to randomly sample 300 households within eight locations representing families caring for orphaned and separated children in the UG County. In order to obtain a representative sample of households caring for orphans in UG County, the project utilized three sampling arms: cash-transfer (CT) households, non-cash transfer households from the same sub-Location (SSL), and non-cash transfer households from a different sub-Location (DSL). The CT program is a government social support initiative that provides regular and predictable (unconditional) cash transfers to poor households taking care of orphans and vulnerable children. The main objective of the CT-OVC program is to encourage fostering and retention of OVC within their families and communities as well as to enhance their human capital development [36]. The CT program targets sub-locations that are the most socioeconomically deprived. Sub-Locations are administrative boundaries within locations and are headed by an Assistant Chief. 100 households were sampled from each category (CT, SSL, and DSL) and weighted to reflect the number of households required per location based on the number of households in each Location caring for orphaned children as provided by the local officials including the DCO, to ensure appropriate distribution.

The DCO oversees the government CT program and provided the study lists of households receiving the government subsidy in each location. For non-CT households, Assistant Chiefs and Village Elders drew up lists of all the households in their villages and sub-Locations caring for orphaned and/or separated children. The lists contained the names of the head of household, their national ID number where available, telephone number where available, the village in which they live, the number of children in the household, and the number of orphaned children in the household. These lists became the sampling frame for the random selection of non-CT (SSL and DSL) households to invite as per the sampling strategy just described.

In total from the three sampling arms there were 2181 households identified; 1370 from the non cash-transfer arm, and 811 from the CT arm respectively. These lists became the sampling frame for the random selection of SSL, DSL and CT households.

Eligible households were required to be caring for orphaned and/or separated children but may also be caring for their own biological children. In order not to ‘single out’ the orphaned child in the household, all children in the household were eligible to participate. In total there were 221 (14.9%) biological children from households caring for orphans whom participated in the study. Households were recruited following extensive community consultations [35,37] and approached individually by Community Health Workers. Consenting, registration, enrolment and all individual study procedures for recruited households took place at the central OSCAR clinic located at Moi Teaching and Referral Hospital (MTRH) in Eldoret.

#### Institutional care environments

Under the Kenyan Children Act (2001), orphanages and other institutions serving orphans are called CCI’s (i.e. children’s homes) if they are able to accommodate ≥20 children [38]. All such institutions being subject to the Kenyan Children Act (2001), located within UG county boundaries, were eligible for recruitment into the study. The UG County Children’s Department maintains a list of registered and unregistered institutions, and has monthly meetings with them in the UG Children’s Services Forum. Two methods were used to identify and recruit CCI’s to participate in the project. First the project utilized the lists of registered CCI’s maintained by the UG Children’s Department and contacted them with a formal letter of introduction from the DCO. Secondly, snowball sampling techniques were used with community members and other stakeholders to identify and contact non-registered CCI’s. The OSCAR project became a member of the UG Children’s Services Forum and was given the opportunity to discuss the research project with forum members. Support was sought from the forum members and the project hoped to identify and sample all eligible CCI’s. The CCI’s were instrumental in identifying names and locations of other CCI’s to the project that could be approached and introduced to the project. In total, there were 21 CCI’s identified in UG County through the two strategies that the project wanted to recruit. For those not able to attend the Forum meeting, we arranged individual meetings with them and/or their Boards of Directors to discuss the study. Of 21 identified CCI’s in the UG County that were contacted, 20 agreed to participate, of whom one was ineligible (they cared for physically and mentally disabled children, not orphans). The project arranged appointments to visit the 19 CCI’s that agreed to participate to facilitate enrolment and assessments of children on their premises. All study procedures for the children in CCI’s took place *in situ* at the institution. All children including the biological offspring of CCI personnel living in the institution (e.g. children of so-called House Parents) were eligible to participate in order not to ‘single out’ the orphaned children. In total there were 51 (3.7%) biological offspring of CCI personnel who participated in the study.

#### Community-based care environments

An initial list of CBO’s was generated based on the knowledge of the project team and through consultation with the UG Children’s Services Forum. CBO’s were contacted and invited to participate in a one-time survey that would describe the services and care they provide for children. Additional CBO’s were contacted through snow-ball sampling by inquiring with CBO directors if they knew of any other CBO’s providing services to orphaned, separated or vulnerable children in Eldoret town that we could contact to participate. In total, there were 8 CBO’s identified in Eldoret through the two strategies that the project wanted to recruit; 7 agreed to participate and one declined.

We also purposively invited the Academic Model Providing Access to Healthcare (AMPATH) [34] Orphaned and Vulnerable Children’s (OVC) Program to provide information regarding their large-scale community-based care program that supports OVC through AMPATH sites in western Kenya.

More details of recruitment and sampling procedures is available elsewhere [35].

### Measures and sources of data

The present analysis utilizes two levels of data: 1) household and facility level data from 300 households, 19 CCI’s and 7 CBO’s that characterized the care environment and 2) individual level data from participants living in households (n = 1481) and CCI’s (n = 1390) through a clinical encounter.

### Household & facility level data

Household and facility level data was collected through a standardized site assessment to ascertain the characteristics of orphaned and separated children’s care environments. The assessment consisted of 12 sections that covered general characteristics, children in residence, resources, shelter characteristics, guardian characteristics, living and sleeping arrangements, food and meals, material, emotional and psychological needs, policies, family linkages, and household food security. The full site assessment is available as an additional file (Additional file 1 and Additional file 2).

### Households

The site assessment was administered in person to heads of households by trained Community Health Workers in their residence. Community Health Workers are residents of the Locations in which they work and have an in-depth knowledge of the households and the cultural context of their communities. As they are representatives of their communities and were introduced by Village Elders, they have developed a rapport and trust with the participants. Due to their trusting relationship and in-depth knowledge of the communities it is likely that respondents are more likely to answer questions honestly. The assessment was validated through random household audits.

### Institutions and CBO’s

The site assessment was administered in person at CCI’s and CBO’s to the institutional Director by the Project Coordinator or Principal Investigator. A modified version of the site assessment was used at CBO’s to capture additional details about the services the organizations provide and omitting questions that pertained to children’s basic material possessions and living conditions as CBO’s typically do not provide residential care.

### Individual level data

Socio-demographic and clinical characteristics were assessed and documented through a standardized clinical encounter form and process. The clinical encounter was intended to be an enhanced well-child ‘check-up’ including a complete history and physical review of systems and symptoms. Educational attendance, orphan status and the child’s primary caregiver, were assessed and documented through a standardized clinical encounter form and process with individual participants. The clinical encounter is administered to children on site at institutions and at the OSCAR’s Health and Well Being Project clinic for those living in households in a private space.

### UN convention on the rights of the child

We purposively selected 10 Articles from the UN Convention on the Rights of the Child that are meaningful to the context of orphan and separated children’s care to understand what extent children’s basic human rights were being respected in institutional vs. community- or family-based care settings. The following Articles were assessed with the measured indicators in brackets: Article 7: The child shall be registered after birth and have the right from birth to a name and nationality, the right to know and be cared for by his or her parents (children have birth certificates, knowledge of family and interaction/regular contact with family); Article 8: Right to preserve identity, including nationality, name and family relations (family connections programs); Article 14: Right to freedom of thought, consciences and religion (policy on participation in religious activities); Article 17: Right to information (presence of books, information and education on HIV prevention, knowledge of parental/family history); Article 19: Right to be protected from all forms of physical or mental violence, injury or abuse, neglect or negligent treatment, maltreatment or exploitation (the use of corporal punishment to enforce discipline); Article 24: Right to health (medical insurance); Article 27: Right to a standard of living adequate for the child’s physical, mental, spiritual, moral and social development (type of shelter and resources, basic material possessions, private cabinet); Article 28: Right to Education (educational attendance); Article 31: Right to rest and leisure, to engage in play and recreational activities (scheduled leisure time, access to toys and games, space or facilities for sports); Article 32: Right of the child to be protected from economic exploitation and from performing any work that is likely to be hazardous or to interfere with the child's education (household tasks children assist with).

### Definitions

Orphan status: A single orphan was defined as a child whose mother *or* father was deceased, and a double orphan as one whose parents were both deceased. A separated child was defined as one for whom at least one parent is completely absent from the child’s life [2].

Basic Material Needs: Children’s basic material needs were defined using UNICEF’s definition that each child have at least one blanket, one pair of shoes and two sets of clothing that are not school uniforms [6].

### Analysis

The analysis was restricted to baseline data collected June 2010-April 2013. Parametric and non-parametric descriptive statistics were employed to summarize both categorical and continuous variables. For continuous variables, mean and median together with standard deviation and inter-quartile range were calculated, respectively. The Chi-Square test was used to test for associations between categorical/dichotomous variables. Fisher’s exact test was also used if some cells had expected value of less than 5. Missing values for selected covariates are reported and were not imputed due to the small numbers of missing data that would not substantially change the interpretation of the results by imputing data. Following validation of eligibility and data cleaning processes, our final sample size at baseline was 2871 (excluding 100 street children and youth). All analyses were conducted using SAS version 9.3.

## Results

**Figure 1:**
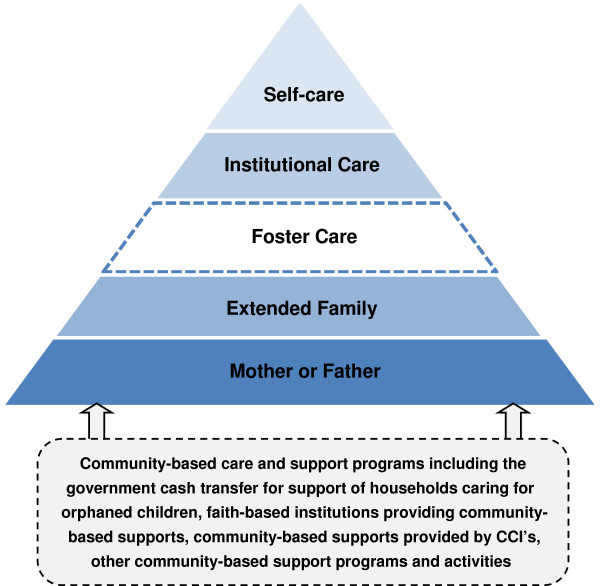
**Proposed hierarchy of models of care for orphaned and separated children.** Models in dotted lines represent opportunities for strengthening family-based care environments.

Included in this analysis were data from 300 households, 19 CCI’s, and 7 CBO’s. In total, 2871 children were enrolled and had baseline assessments done: 1390 in CCI’s and 1481 living in households in the community. Among children in households, 63.2% and 22.1% are single and double orphans/separated children respectively; compared to 43.6% and 53.5% in CCI’s. In total there were 275 (9.2%) non-orphaned children that participated in the study from households and CCI’s. Additional details about the study population have been reported elsewhere [35,39-41]. We identified four broad models of care for orphaned and separated children in UG County, Kenya, including: institutional care, family-based care, community-based care and self-care. The main models of care discussed in this paper include institutional care, family-based care, and community-based care (Table 2). From the results of this paper we have proposed a hierarchy of care models based on those that are existing and opportunities for expansion (Figure 1). Models outlined with dotted lines (community-based care and support, foster care) represent opportunities identified from this work that could strengthen family-based care provision. Self-care, which occurs predominantly among street youth is discussed elsewhere [41].

**Table 2:** Models of care for orphaned and separated children in Uasin Gishu County, Kenya

**Model of Care**	**Description**	**Image of Care Environment**	**Characteristics**	
**Pure CCI**	CCI’s that solely provide long-term residential care to orphaned and separated children.	**Village Style – Pure CCI**	**# of Children in residence** (n)	630
			**Single Orphans** (%)	43.3%
			**Double Orphans** (%)	53.2%
			**Child to caregiver ratio**	8:1
			**Maximum Capactiy of home**	
	• 91% limit admission to children < 10 years of age		Day, median (IQR)	45 (20-145)
**n=11**	• 45% limit admission to double orphans		Night, median (IQR)	43 (20-96)
			**Living Arrangements,** n (%)	
			Village Style	2 (18%)
	• Typically faith-based care environments (82%)		Dormitory	2 (18%)
			Single Family	6 (55%)
			Mixed	1 (9%)
**CCI Plus**	CCI’s that provide home support and facilitate community-based programs to enable orphans to remain living in family-based care, in addition to providing long-term residential care	**Single Family Home - CCI Plus**	**# of Children in residence** (n)	446
			**Single Orphans** (%)	39.8%
			**Double Orphans** (%)	55.5%
			**Child to caregiver ratio**	12:1
			**Maximum Capactiy of home**	
			Day, median (IQR)	62 (50-80)
			Night, median (IQR)	62 (20-80)
**n=6**	• Programs provide school fees to children in the community and psychosocial support for families		**Living Arrangements,** n (%)	
			Village Style	1 (17%)
			Dormitory	2 (33%)
			Single Family	2 (33%)
			Mixed	1 (17%)
**CCI Shelter**	Temporary residential care facilities that are typically a place of last resort for children and youth. Meant to provide temporary care, yet children end up residing long-term as there is a lack of alternatives.	**Dormitory Style - CCI Shelter**	**# of Children in residence** (n)	314
			**Single Orphan** (%)	48.1%
			**Double Orphan** (%)	49.7%
			**Child to caregiver ratio**	14:1
**n=2**			**Maximum Capactiy of home**	
			Day, median (IQR)	158 (65-250)
			Night, median (IQR)	158 (65-250)
			**Living Arrangements,** n (%)	
	• Government facilities		Village Style	0 (0)
	• Shelters for street children		Dormitory	2 (100%)
	• Probation centres		Single Family	0 (0)
	• Court mandated care		Mixed	0 (0)
**Family-based care**	Occurs in the community where a child remains within a family setting	**Typical UG County Rural Household**	**# of Children in residence** (n)	1481
	• Immediate family (surviving parent)			
			**Single orphans** (%)	63.1%
			**Double Orphans** (%)	22.0%
	• Extended Family			
**n=300**	•Foster care (formal and informal)		**Child to caregiver ratio**	3:1
			**Maximum Capactiy of home**	
			Day, median (IQR)	5 (3-6)
			Night, median (IQR)	5 (3-6)
**Community-based care**	Community-based care often occurs through community-based organizations (CBO’s). Community-based care providers enable children to remain in family-based care environments by providing material goods and support service to build a households capacity and ensure they can meet the needs of the children.	**Community-based Organization**	**# of children supported in**	50 (41-74)
			Day Programs	50 (38-65)
			Feeding Program	0 (0-25)
			After school program	4 (1-15)
			Residence	50 (32-60)
**n=7**			Other	
			**# of households assisting**	30 (12-50)

### Overview of models

#### Institutional care

We identified environmental characteristics that distinguished CCI’s into different categories: “Pure CCI’s” (n = 11), “CCI Plus” (n = 6) and “CCI Shelter” (n = 2) that are described in Table 2. We categorized CCI’s to differentiate between those that are in the true sense places of residential care for orphaned and separated children (traditional orphanages here called “Pure CCI’s”) versus those that provide a combination of residential care and community supports (“CCI Plus” such as those that also provide school fees and/or psychosocial support to orphans who continue living with their extended family), versus those that provide mandated care, such as probation and detention centres (“CCI Shelter”). The median number of orphaned and separated children in residence at Pure CCI’s was 40 (IQR: 24–104), 66 (IQR: 47–82) at CCI Plus and 152 (IQR: 45–260) at CCI Shelter institutions. The median number of adults living full time at Pure CCI’s was 5 (IQR: 4–10), 6 (IQR: 4–8) at CCI Plus, and 11 (IQR: 10–12) at CCI Shelter institutions.

In general, most CCI’s were registered institutions (79%) and governed by volunteer boards of directors/trustees (74%) (Table 3). The median number of orphaned or separated children in residence at CCI’s was 51 (IQR: 25–94), with a child to caregiver ratio of 10:1 (Total Children in Residence: Total Adults Living Full-time in Facility). Almost all CCI’s had age criteria for admission (84%), with the majority limiting admission to children aged 12 years and younger (n = 14, 74%).

**Table 3:** General characteristics of care environments in Uasin Gishu County, Kenya

**Characteristics**	**Institutional care**				**Family-based care N = 300**	**Community-based care N = 7**
	**Children’s charitable constitutions**					
	**N = 19**					
	**Total n (%)**	**CCI pure n = 11 n (%)**	**CCI plus n = 6 n (%)**	**CCI shelter n = 2 n (%)**	**n (%)**	**n (%)**
Registered	15 (79)	9 (82)	4 (67)	2 (100)	-	6 (86)
Unregistered	4 (21)	2 (18)	2 (33)	0 (0)	-	1 (14)
**Environment Type**						
Secular	3 (16)	1 (9)	2 (33)	0 (0)	-	4 (57)
Faith-based	12 (63)	9 (82)	3 (50)	0 (0)	-	4 (57)
NGO	2 (10.5)	1 (9)	1 (17)	0 (0)	-	2 (29)
Governmental	2 (10.5)	0 (0)	0 (0)	2 (100)	-	0 (0)
**Governance Structure**						
Village chief or elders	0 (0)	0 (0)	0 (0)	0 (0)	1 (<1)	0 (0)
Head of household	0 (0)	0 (0)	0 (0)	0 (0)	294 (98)	0 (0)
Private individual	2 (10.5)	1 (9)	1 (17)	0 (0)	3 (1)	0 (0)
Remunerated board of directors/trustees	2 (10.5)	2 (18)	0 (0)	0 (0)	0 (0)	0 (0)
Volunteer board of directors/trustees	14 (74)	8 (73)	5 (83)	1 (50)	0 (0)	7 (100)
Other advisory committee	1 (5)	0 (0)	0 (0)	1 (50)	0 (0)	0 (0)
*Missing data*					2	
**Admission Criteria for the Household/Institution**						
Family member or child of friend	0 (0)	0 (0)	0 (0)	0 (0)	121 (40)	0 (0)
Age criteria	16 (84)	11 (100)	3 (50)	2 (100)	0 (0)	6 (86)
HIV +	1 (5)	1 (9)	0 (0)	0 (0)	2 (<1)	0 (0)
HIV –	5 (26)	3 (27)	2 (33)	0 (0)	0 (0)	0 (0)
Any orphan	5 (26)	3 (27)	2 (33)	0 (0)	10 (3)	2 (29)
Double orphan	6 (32)	5 (45)	1 (17)	0 (0)	3 (1)	1 (14)
On/Of the street child	2 (10.5)	0 (0)	1 (17)	1 (50)	0 (0)	3 (43)
Abused or abandoned	5 (26)	4 (36)	0 (0)	1 (50)	2 (<1)	3 (43)
Other^‡^	5 (26)	2 (25)	2 (33)	1 (50)	0 (0)	0 (0)
**Director or Head of Household Characteristics**						
Age, median (IQR)	49 (42–60)	47 (38–57)	47 (42–60)	70 (70)	48 (37–57)	45 (39–58)
**Highest level of education**						
None	0 (0)	0 (0)	0 (0)	0 (0)	62 (21)	0 (0)
Primary	0 (0)	0 (0)	1 (17)	0 (0)	162 (54)	0 (0)
Secondary	3 (16)	2 (17)	3 (50)	1 (50)	61 (20)	3 (43)
Vocational	8 (42)	5 (42)	2 (33)	0 (0)	1 (<1)	0
College/University	8 (42)	5 (42)	0 (0)	1 (50)	1 (<1)	4 (57)
Other	0 (0)	0 (0)	0 (0)	0 (0)	11 (4)	
*Missing*					2	
**Legally Mandated Guardian of Children**						
Yes	18 (95)	11 (100)	5 (83)	2 (100)	188 (64)	-
No	1 (5)	0	1 (17)	0	107 (36)	-
*Missing*					5	
**Guardian Kenyan**						
Yes	16 (84)	9 (82)	5 (83)	2 (100)	294 (>99)	7 (100)
No	3 (16)	2 (18)	1 (17)	0 (0)	1 (<1)	0 (0)
*Missing*					5	

^‡^includes: 2 court ordered, 1 HIV-exposed, 1 not on drugs, 1 poverty.

We recognized various styles of living arrangements within the CCI’s that we classified into the following categories: single family-homes (n = 8), dormitory style (n = 6), village style (n = 3), and mixed arrangements (n = 2) (a combination of styles) (images Table 2). In all living arrangements, boys and girls slept separately and 74% of homes clustered children based on their age.

Dormitory style living arrangements have large sleeping quarters that house many children together in one room with 67% of CCI’s separating children into dorms based on age. The facilities have separate communal living and eating areas. Guardians typically stay in their own room within the same building or in another building within the compound.

Village style homes emulate typical Kenyan communities and villages with clusters of houses (circular or square) grouped as family units. These homes have 1 house mother or 2 house parents (mother and father) that look after their children in a family unit. Typically a family unit consists of 12–20 children living with their house parent(s). Most commonly, children within a family unit have their own living space and eat together as a family with their house parent(s).

Single family homes have children in care living within a large family unit under one roof with the institution’s Directors living within the home acting as the mother and father of all of the children. This style emulates single family households with communal living areas and separate rooms housing small groups of children.

### Community-based care

Community-based care refers to support programs and services offered to children and households that typically enable children to remain in family-based care environments. Community-based care often occurs through CBO’s; however, CCI Plus residences and large scale specialized programs such as the AMPATH OVC program facilitate community-based care within UG County.

CBO’s provide support to orphaned and vulnerable children and households through various programs in their respective communities. These organizations have strong community linkages and all interact regularly with community members through forums, open houses, and meetings with guardians. CBO’s in UG County offered a diversity of programs for orphaned and vulnerable children. Over half of the CBO’s offered day programs and the majority operated on both weekdays and weekends (Table 4). The median number of children attending day programs was 50 (IQR: 41–74). Three of the CBO’s specifically provide services to street-involved children and youth, two have specific mandates to reach out to people living with HIV/AIDS and supporting AIDS orphans, while others cater to all OVC in need. All CBO programs indicated they supported school fees, uniforms, and school supplies, offered a feeding program and provided emotional support. In some cases, CBO’s provided temporary or emergency shelter and had children in residence. Five of the seven CBO’s identified were providing short- or long-term residence to children in need, with the median number of children in residence being 4 (IQR: 1–15).

**Table 4:** Operational Characteristics of Community-based organizations

**Characteristics**	**CBOs**
	**N = 7**
	**n (%)**
**Hours of operation**	
Days only	4 (57)
Days and nights	3 (43)
Weekdays only	2 (29)
Weekends only	0 (0)
Both	5 (71)
**Participation initiated by**	
Child	5 (71)
Guardian	2 (29)
Organization	6 (86)
Community referral	5 (71)
**Guardians know children attending**	
Yes	4 (57)
No	0 (0)
Unsure	3 (43)
**Permission of guardian required**	
Yes	3 (43)
No/sometimes/unsure	4 (57)
**Number of paid staff** median (IQR)	3 (1–9)
**Number of volunteers** median (IQR)	4 (3–5)
**Material assistance provided to participants**	
Money	2 (29)
School fees	7 (100)
School uniforms or other school needs	7 (100)
Mattresses and/or blankets	5 (71)
Household repairs	1 (14)
Bed-nets	4 (57)
Transportation	2 (29)
Food Items	5 (71)
Seeds or agricultural inputs	0 (0)
**Additional forms of assistance provided**	
Emotional support	7 (100)
Feeding program	7 (100)
Sanitary pads	5 (71)
Medical assistance	4 (57)
Social work	6 (86)
Informal education/vocational	5 (71)
Day-care pre-school aged children	5 (71)
Emergency shelter	2 (29)
Long-term shelter	5 (71)

The AMPATH OVC program is a large scale community-based care program that provides a holistic and multi-disciplinary approach to support to strengthen the capacity of families and communities to care for and protect OVC in western Kenya. A total of 9,670 households and 22,974 OVC receive support with the majority (70%) residing in UG County. The program accepts all orphans, vulnerable HIV-positive or negative children, and those who have been abused or abandoned. Participation in the program may be initiated by guardians, through AMPATH, or in the community. OVC are enrolled and monitored in the program until they are 17. AMPATH OVC staff work with families to determine their needs using comprehensive household and individual assessments. Material support and social services are provided to the household until they stabilize including: school fees and supplies, household repairs, mattresses/blankets, agricultural inputs, food items, transportation, vocational training, emergency shelter, emotional support, social work and medical assistance. The program also facilitates family linkages through attempted repatriation and ensuring children know who their family history. Once a family stabilizes, households are monitored by an OVC Community Health Worker or Social Worker to evaluate the impact of the program’s interventions and to ensure that the children’s and family’s needs have been addressed.

### Family-based care

**Figure 2:**
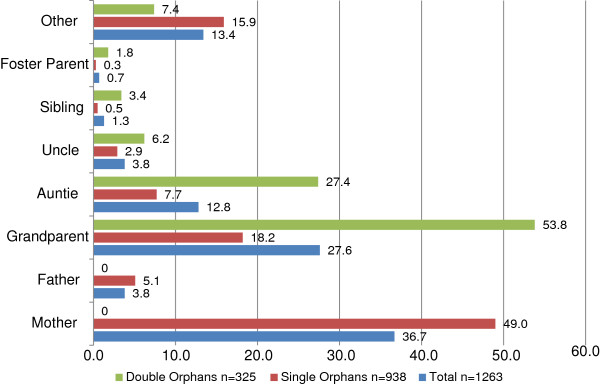
**Percentage of orphaned and separated children living with specified primary caregiver from 300 family-based environments.** (Note: additional adults may be living in the household)

Family-based care is that which occurs in the community and may take on a number of forms including: care by a surviving parent, extended family care, and foster care. The median number of orphaned and separated children in family-based care was 5 (IQR: 3–6) with a median of 1 (IQR: 1–2) caregiver living full-time in the household. Of children in family-based settings not living with both their mother and father, 40% remained in the care of a surviving parent, 44% in extended families and less than 1% in foster care. Very few fostering arrangements occur in UG County; foster care may be formal (legally mandated guardian) or informal, as occurs in cases when a non-relative takes on caring for orphaned or separated children, usually a neighbour or family friend. Figure 2 demonstrates family-based care giving arrangements for both single and double orphans. Single orphans most often remain with the mother (49%), followed by a grandparent (18%) or aunt (8%). Double orphans are typically cared for by a grandparent (54%) or aunt (27%).

### Ability of models to uphold children’s rights

Table 5 details indicators of children’s rights being upheld in institutional care, family-based care and through community-based care programs for orphaned and separated children based on the specific indicators and selected rights in Table 1.

**Table 5:** Indicators of children’s rights being upheld in care environments in Uasin Gishu County, Kenya

**Characteristics**	**Institutional care**				**Family-based care N = 300**	**Community-based care N = 7**
	**Children’s charitable institutions**					
	**N = 19**					
	**Total n (%)**	**CCI pure n = 11 n (%)**	**CCI plus n = 6 n (%)**	**CCI shelter n = 2 n (%)**	**n (%)**	**n (%)**
**ARTICLE 27: RIGHT TO ADEQUATE STANDARD OF LIVING**						
**Type of shelter**						
Temporary/semi-permanent	1 (5)	0 (0)	0 (0)	1 (50)	286 (96)	3 (43)
Permanent	18 (95)	11 (100)	6 (100)	1 (50)	11 (4)	4 (57)
*Missing*					3	
**Does the shelter have electricity**						
Yes	17 (89)	9 (82)	6 (100)	2 (100)	28 (9)	6 (86)
No	2 (11)	2 (18)	0	0	268 (91)	1 (14)
*Missing*					4	
**Drinking water source**						
Water piped into home	9 (47)	7 (64)	1 (17)	1 (50)	21 (7)	5 (71)
Borehole/well	8 (42)	4 (36)	3 (50)	1 (50)	220 (73)	3 (43)
Public standpipe	2 (11)	0 (0)	2 (33)	0 (0)	50 (17)	0 (0)
River, stream, pond, lake, ditch,	0 (0)	0 (0)	0 (0)	0 (0)	36 (12)	0 (0)
**Basic Material Needs**^**‡ **^**(combined UNICEF)**						
Yes	18 (95)	11 (100)	6 (100)	1 (50)	50 (17)	-
No	1 (5)	0 (0)	0 (0)	1 (50)	242 (83)	-
*Missing*					8	
**Each child has own bed/mattress**						
Always	12 (63)	7 (64)	3 (50)	2 (100)	3 (1)	-
Sometimes	2 (11)	1 (9)	1 (17)	0	19 (6)	-
No	5 (26)	3 (27)	2 (33)	0	276 (93)	-
*Missing*					2	
**Number of blankets each child has**						
Not all children have at least 1	0 (0)	0 (0)	0 (0)	0 (0)	226 (77)	-
1	7 (37)	4 (36)	1 (17)	2 (100)	50 (17)	-
>1	12 (63)	7 (64)	5 (83)	0 (0)	19 (6)	-
*Missing*					5	
**Bed nets on each bed/mattress**						
Yes	10 (53)	7 (64)	3 (50)	0	100 (34)	-
No	9 (47)	4 (36)	3 (50)	2 (100)	194 (66)	-
*Missing*					6	
**Each child has private cabinet/box/drawer**						
Yes	11 (58)	8 (73)	2 (33)	1 (50)	17 (6)	-
No	8 (42)	3 (27)	4 (67)	1 (50)	281 (94)	-
*Missing*					2	
**Each child has at least one pair of shoes**						
Yes	18 (95)	11 (100)	6 (100)	1 (50)	249 (84)	-
No	1 (5)	0 (0)	0 (0)	1 (50)	49 (16)	-
*Missing*					2	
**Each child has at least 2 pairs of clothing**						
Yes	19 (100)	11 (92)	6 (100)	2 (100)	259 (86)	-
No	0 (0)	0 (0)	0 (0)	0 (0)	41 (14)	-
**Number of uniforms each school-going child has**						
Not all children have complete uniform	0	0	0	0	76 (26)	-
1	7 (39)	5 (45)	2 (33)	1 (50)	211 (71)	-
>1	11 (61)	6 (55)	4 (67)	1 (50)	10 (3)	-
*Missing*	1				3	
**ARTICLE 17: RIGHT TO INFORMATION**						
Books Available	14 (74)	9 (82)	4 (67)	1 (50)	41 (14)	6 (86)
HIV Education	14 (74)	7 (64)	5 (83)	2 (100)	278 (93)	4 (57)
Knowledge of family history	13 (81)	7 (64)	4 (80)	2 (100)	190 (67)	3 (60)
*Missing*	3	2	1		17	2
**ARTICLE 24: RIGHT TO HEALTH**						
Medical insurance	1 (5)	1 (8)	0 (0)	0 (0)	16 (5)	-
Missing					7	
**ARTICLE 31: RIGHT TO LEISURE**						
Toys available	11 (58)	6 (55)	5 (83)	0 (0)	4 (>1)	3 (43)
Games available	15 (79)	8 (73)	5 (83)	2 (100)	3 (1)	5 (71)
Scheduled leisure time	14 (74)	8 (73)	5 (83)	1 (50)	26 (9)	6 (86)
Space or facilities for sports	14 (74)	7 (64)	5 (83)	2 (100)	237 (79)	5 (71)
**ARTICLE 32: RIGHT TO PROTECTION FROM EXPLOITATION**						
**Household tasks children assist with**						
Cooking	12 (63)	6 (55)	5 (83)	1 (50)	252 (84)	4 (57)
Child care	10 (53)	4 (36)	5 (83)	1 (50)	105 (35)	2 (29)
Water collection	3 (16)	2 (18)	1 (17)	0 (0)	274 (91)	2 (29)
Firewood collection	1 (5)	1 (9)	0 (0)	0 (0)	202 (67)	1 (14)
Food gathering	1 (5)	0 (0)	1 (17)	0 (0)	50 (17)	2 (29)
Income generating activities	0 (0)	0 (0)	0 (0)	0 (0)	17 (6)	1 (14)
Animal care	8 (42)	2 (18)	4 (67)	2 (100)	77 (26)	2 (29)
Washing clothes/dishes/cleaning	12 (63)	7 (64)	4 (67)	1 (50)	26 (9)	0 (0)
Other	1 (5)	1 (9)	0 (0)	0 (0)	1 (<1)	0 (0)
None	0 (0)	0 (0)	0 (0)	0 (0)	6 (2)	0 (0)

^‡^Unicef definition basic material needs met (defined as having one blanket, one pair of shoes, and two sets of non-school clothes.

### Right to birth registration (article 7) and identity and family relations (article 8)

A child has the right to preserve his or her identity, including nationality, name and family relations. Very few children in institutional care (20%) and family-based care (21%) had birth certificates. A high number of institutions (n = 17) and households (n = 235) reported major difficulties with obtaining birth certificates for resident children because of the government requirement that one or both biological parent’s identification be appended to the application. Most had birth certificate applications pending with the government registrar of births and deaths, and were unsure how to answer the question. Most CCI’s indicated they had a policy or program on family connections (n = 17, 89%), typically in the form of regular contact with the family (n = 15, 79%), attempted repatriation (n = 8, 42%), and ensuring the children know who their parents are/were (n = 7, 37%). CCI’s indicated the majority of children know their parental/family history (n = 13, 81%). Similarly, 71% of CBO’s had a program on family connections, most frequently in the form of attempted repatriation (n = 5, 71%), regular contact with the family (n = 4, 57%) and family support programs (n = 4, 57%).

### Right to freedom of thought, consciences and religion (article 14)

A child has the right to explore and express different views. This is recognized by the Government of Kenya through the approved religious curriculum for educational facilities (i.e. schools) which offer students the choice of whether to study Christian, Muslim, or Hindu religion. Most places of institutional care (63%) in our study and 82% of Pure CCI’s indicated they were faith-based environments. The majority (84%) of institutions offered daily religious education, and 58% had compulsory religious education. Similarly, 57% of community-based care providers consider themselves a faith-based environment; with 29% having compulsory religious education and 43% offering daily religious education. In comparison, only 11% of family-based care environments had compulsory religious education for their children (missing 18 responses).

### Right to education (article 28) & information (article 17)

The majority (97%) of school-aged children in institutional and family-based care had ever gone to school. However, orphaned children in institutional care were significantly more likely to be currently enrolled (96.3% vs. 94.4% p < 0.05) and attending school (96.4 vs 94.1% p < 0.05) in comparison to those in family-based care. Within family-based care environments there was no significant difference in school attendance between orphans and non-orphans. Books were commonly available in CCI’s (74%) and CBO’s (86%) and not commonly available in households (14%). The majority of households (93%) reported they provided HIV prevention education in comparison to 74% of CCI’s and 57% of CBO’s.

### Right to be Protected from all Forms of Violence (Article 19)

A child has the right to be protected from all forms of physical or mental violence, injury or abuse, neglect or negligent treatment, maltreatment or exploitation. Most family-based care environments indicated they enforced discipline utilizing corporal punishment (78%), with fewer utilizing discussions with children regarding their behaviour (35%), and scolding (8%). In contrast, in institutional care the most common methods of enforcing discipline for children were using discussions with children regarding their behaviour (79%), withholding privileges (47%), assigning additional chores (26%) and corporal punishment (26%). Similarly, at CBO’s the most common methods of enforcing discipline for children were using child psychology (71%), additional chores (57%), and withholding privileges (43%), with very few utilizing corporal punishment (14%).

### Right to adequate standard of living (article 27) & basic material needs

A child has the right to a standard of living adequate for the child’s physical, mental, spiritual, moral and social development. To assess standards of living we inquired about a variety of socio-economic indicators including shelter characteristics, sources of income, and basic material goods. Almost all (96%) families live in temporary or semi-permanent houses, while 95% of CCI’s and 57% of CBO’s have permanent structures. Only 9% of family-based care environments have electricity in contrast to 89% of institutions and 86% of CBO’s. Institutions (47%) were significantly more likely (Odds Ratio (OR) = 9.66, 95% Confidence Interval (CI): 3.51 – 26.61) to have water pumped into the home than family-based settings (7%). Many families indicated they had no external support (36%), and those who did report external support specified that they typically received this from family (39%) and government (33%), the latter through the government cash transfer program for orphaned children. Very few families received support from individual donors (16%) and religious institutions (2%). In comparison, all CBO’s and CCI’s received external support with the majority from individual donors (100% and 84%, respectively), religious institutions (86% and 42%), and non-governmental organizations (57% and 47%). CCI’s received most funding from external sources, as 42% indicated they had no other sources of income. Most commonly households received income from farming (53%), casual labor (27%), and selling vegetables (19%). Only 2% of households received income through formal employment and 2% indicated they received income from brewing alcohol. CCI’s that had other sources of revenue typically received income from farming (37%). CBO’s that indicated they had additional sources of income received funds from operating a school (29%), farming (14%) and livestock (14%). Almost all CCI’s owned land (n = 18, 95%) with 79% owning greater than 1 acre. In contrast, 71% of households owned land, with 37% owning greater than 1 acre. Similar numbers of CCI’s, households and CBO’s indicated they grew cash crops, 32%, 32% and 29% respectively. Food crops were grown by 89% of CCI’s in comparison to 67% of households and 57% of CBO’s.

To assess the capacity of family-based and institutional environments to provide children basic material needs we used UNICEF’s definition that each child have at least one blanket, one pair of shoes and two sets of clothing that are not school uniforms. Children in institutional care were significantly more likely to have their basic material needs met (95%) in comparison to those in family-based care (17%) (p < 0.0001).

## Discussion

Our findings demonstrate that there are many models of care for orphaned and separated children and that each plays a valuable and important role in the response to the orphan crisis in sub-Saharan Africa. This is supported by what others have found in Malawi and South Africa [20,24]. However, we identified several important differences in institutional, family- and community-based care environments with respect to their ability to uphold children’s rights and provide basic material needs, while identifying opportunities to strengthen family-based orphan care.

First, family-based care environments in UG County are significantly less likely to be able to provide for children’s basic material needs than CCI’s and may not be able to provide a standard of living adequate for the child’s physical, mental, spiritual, moral and social development. This is consistent with other research from sub-Saharan Africa indicating that extended families are stretched and unable to meet their care-taking expectations including providing, food, clothing and other basic needs for children [7,9,15,42]. Second, Kenya’s constitution prohibits corporal punishment [43]; yet the majority of families (77%) and a quarter of institutions report using this method of enforcing discipline. The use of corporal punishment is an ineffective means of discipline, is linked to child abuse, may have long-term negative effects on the child [44-47], and violates the Convention on the Rights of the Child Article 19 and 37 [30] and therefore is a fundamental breach of children’s human rights [48]. Third, despite reports in other sub-Saharan African settings stating that children in institutional care lack links to family, and community [26,27,49], CCI’s and CBO’s in UG County worked to facilitate family connections through their programs and ensuring that children in their care have knowledge of and where possible, contact with, their families. Fourth, very few children in both institutional and family-based care have birth certificates and therefore are lacking their right to their identity. Children without birth certificates have difficulties accessing education, obtaining identity documents and other items necessary to transition to successful adulthood [27,42]. Fifth, while religion plays a central role in Kenyan culture [50], the rights of children to freedom of thought, conscience and religion may be infringed upon in CCI’s as the majority are faith-based environments and over half have compulsory religious education. Lastly, the majority of institutional care environments limit admission to children below the age of 12 and therefore orphaned/separated adolescents are lacking care options when no extended family is able to take on this responsibility and are being subject to discrimination due to their age. Yet, these institutional care environments are an important place of last resort for orphans and non-orphans alike as it has been documented that over 90% of non-orphaned children living in CCI’s were admitted due to maltreatment and the majority of orphans due to extreme destitution [51].

We recognized several opportunities to strengthen family-based models of care. First, there lacked households providing foster care in UG County. Foster care by non-relatives may be a viable solution to enable children to remain living in family-based care environments that has been overlooked, yet plays a role in many other sub-Saharan African countries [42]. Second, CBO’s and CCI Plus models of care should be expanded and augmented, as these programs provide support to families caring for orphans and enable children to remain in family-based settings. Augmenting programs providing school fees, uniforms, psychosocial support, and economic development opportunities can build capacity with families to ensure they can meet the needs and protect the rights of children in their care. Lastly, religious institutions provide extensive support to institutional care providers; yet they have not reached out into the communities to support households caring for orphaned and separated children which represent a missed opportunity to ensure children remain in family-based care. Additionally, findings from other studies indicate that expanding the government Cash Transfer to Orphans and Vulnerable Children program may have the ability to build capacity for family-based care and protect orphaned children living in poor households [36,52]; as children living in households receiving this transfer in UG County have improved school attendance, nutritional status, and future outlook on life [39].

There are several strengths to our findings. The first is that these data come from a well-defined geographic area, and are representative of orphaned and separated children in Uasin Gishu County, Kenya. Second, we were able to randomly sample households to obtain a representative sample and reduce selection bias. Third, we comprehensively recruited and assessed all CCI’s and a large proportion of CBO’s providing care in UG County. Lastly, we used empiric methods to measure the ability of different care environments to uphold children’s rights.

There may be limitations to our findings. Many of the outcome measures are self-reported and therefore may be subject to various kinds of bias, including reporting bias such as interviewer or social desirability bias. We tried to minimize these potential biases through the deployment of Community Health Workers to conduct the household level data collection and by using unannounced household audits. However, highly sensitive data concerning issues such as corporal punishment may still be biased by self-report and validated measures of discipline were not utilized in the data collection instrument. Although the site assessment was administered to the head of household or facility Director, children were not asked the same questions and may have responded differently to questions regarding their basic material possessions, corporal punishment or other characteristics of their care environment. However, for many of the questions posed in the site assessment the head of household or facility Director would be able to provide the most accurate answer regarding the care environment’s characteristics. Additionally, because the facility and household level data were collected on the unit of the household/facility and not per individual child, we were not able to disaggregate the data separately for the 275 (9.2%) of non-orphaned children living in the same care environments as the orphans. These data may also be subject to random misclassification bias if there were errors in recording or data entry. Lastly, because there are so few registered foster families, we were unable to systematically invite foster families to participate in the study.

## Conclusion

In conclusion, these findings demonstrate that each model of care has strengths and weaknesses. Family-based care is clearly essential; however, households require significantly increased support to adequately care for children and ensure children’s rights are being upheld. CBO’s and religious institutions have the potential to assist families struggling to provide care to orphans. CCI’s and CBO’s are important care models and both could expand their community-based support programs to support children and families in need. All of these models play a key role in response to the orphan crisis. While it is ideal that children remain in family-based care, CCI’s are needed as a last resort in the hierarchy of care and act as a safety net protecting the most vulnerable from falling into self-care on the streets. While the number of orphans continues to increase in sub-Saharan Africa, there is a need to take a ‘both-and’ approach rather than an ‘either-or’ one to care for and support the immense number of orphaned and separated children in need of care on the continent.

## Competing interests

The authors declare they have no competing or financial interests.

## Authors’ contributions

LE and PB led the development of the manuscript. LE and JK performed the statistical analysis. DA & PB conceived, designed and supervised the study. AK, LA, DA, WN, PG, SA & RV assisted in writing the manuscript and LA, DA, WN, PG, SA & RV contributed to the study design. All authors read and approved the final manuscript.

## Pre-publication history

The pre-publication history for this paper can be accessed here:

http://www.biomedcentral.com/1472-698X/14/9/prepub

## Supplementary Material

Additional file 1 Site Assessment (Community-Based Organizations). Click here for file

Additional file 2 Site Assessment (Households and Institutions). Click here for file
