# Supplementary material for: Models of care for orphaned and separated children and upholding children’s rights: cross-sectional evidence from western Kenya
Source: BMC Int Health Hum Rights. 2014 Apr 1;14:9. doi: 10.1186/1472-698X-14-9 (PMC4021203; doi:10.1186/1472-698X-14-9)
Supplement: Additional file 1 — Site Assessment (Community-Based Organizations). [file 1472-698X-14-9-S1.doc]

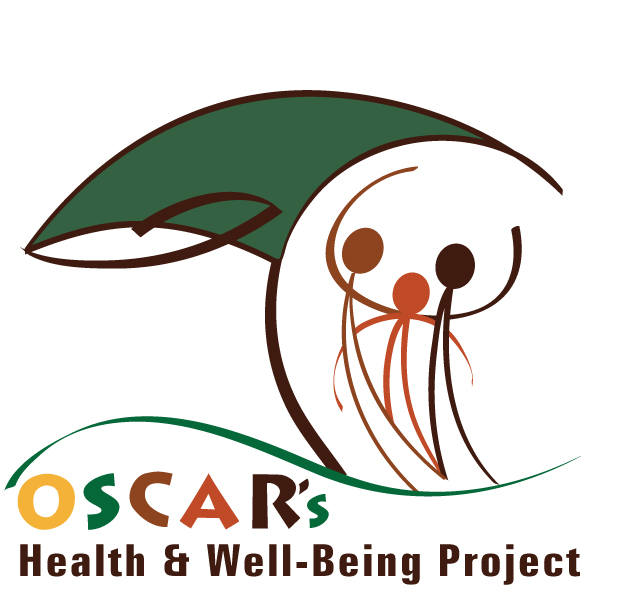


***Site Assessment (Community-Based Organizations)***

**Facility Code: _________________**

**Date: ________________________________**

**Person administering questionnaire: _______________________________________________**

***These questions are to be asked of the Director or In-Charge.***

***1.0 Contact Information:***

| **1.1**  **Name of Director (specify role):** |  |
| --- | --- |
| **1.2**  **Name of Facility:** |  |
| **1.3**  **Location, Sub-Location, Village:** |  |
| **1.4**  **Contact phone numbers:** |  |
| **1.5**  **Email address and or website:** |  |
| **1.6**  **GPS Coordinates:** |  |

**2.0 GENERAL**

| **Question** | **Answer** |
| --- | --- |
| **2.1**  **Type of Environment**  **(tick all that apply)** | ___**Registered Community-Based Organization (CBO)**  **Please select the applicable sub-category**  ___ Faith-based CBO  ___ Secular CBO  ___ International non-governmental organization run CBO  ___ Other (specify):____________________________  ___**Registered association (specify):_________________**  ___**Unregistered**  **Please select the applicable sub-category**  ___ Faith-based  ___ Secular  ___ International non-governmental organization  ___ Other(specify):____________________________ |
| **2.2**  **When did the organization begin functioning?** | Day:__________  Month: ________  Year: __________ |
| **2.3**  **Governance Structure**  **(tick all that apply)** | ___Village Chief or Elders  ___ Head of household  ___Private individual  ___Single Remunerated In-Charge/ Director (no Board)  ___Single Volunteer In-Charge/ Director (no Board)  ___Remunerated Board of Directors/Trustees  ___Volunteer Board of Directors/Trustees  ___Other Advisory Committee  ___Other (specify): _________________________________ |
| **2.4**  **Hours and days of operation** | **Hours**  ___Days only  ___Days and nights  **Days of operation**  ___Weekdays only  ___Weekends only  ___All the time  ___Other (specify):____________________________________ |
| **2.5 Does the organization regularly interact or collaborate with the community?** | ___Yes ___Unsure  ___No ___Refuse to answer  If ***Yes***, in what capacity: |

**Comments:** ______________________________________________________________________________________________________________________________________________________________________________________

___________________________________________________________________________________________

| **Question**  **3.0 CHILDREN PARTICIPATING** | **Answer** |
| --- | --- |
| **3.1**  **How many children does the organization serve? (please indicate # for each category and total #. If not applicable indicate by N/A)** | **Children in day program**  Cumulative: _____  Active (within past 12 months): ____  **Children in after school program**  Cumulative: _____  Active (within past 12 months): ____  **Children in residence**  Cumulative: _____  Active (within past 12 months): ____  **Children in feeding program**  Cumulative: _____  Active (within past 12 months): ____  **Children in other programs**  Cumulative: ____  Active (within past 12 months): **____**  **Total**  Cumulative: _____  Active (within past 12 months): ____ |
| **3.2**  **How many families/households does the organization assist?** | Cumulative: _____  Active (within past 12 months): ____ |
| **3.3**  **How many children are there in residence? (indicate #s for each category)** | Aged 0-4 Boys:___ Girls: ___  Aged 5-10 Boys: ___ Girls: ___  Aged 11-17 Boys: ___ Girls: ___  Aged 18+ Boys: ___ Girls: ___ |
| **3.4**  **How long do children typically stay in residence at your organization?**  **(please indicate # of days, or months or years)** | Days: ___  Months:___  Years:___ |
| **3.5**  **How many of the participating children are in school? (indicate #s)** | Aged 5-10 Boys: ___ Girls: ___  Aged 11-17 Boys: ___ Girls: ___  Aged 18+ Boys: ___ Girls: ___ |
| **3.6**  **Who initiates child participation in the organization? (tick all that apply)** | ___ Child initiated  ___ Guardian initiated  ___ Organization initiated through outreach  ___ Community referral  ___ Other (specify):______________________________ |
| **3.7**  **Do guardians typically know their children are coming here?** | ___Yes ___Unsure  ___No ___Refuse to answer |
| **3.8**  **Is permission of guardian required for children to participate?** | ___Yes ___Unsure  ___No ___Refuse to answer |
| **3.9**  **Does organization have a mechanism for communicating with guardians?** | ___Yes  ___No  **If yes: How?**  ___Telephone numbers  ___Household visits  ___Post  ___Email  ___Other (specify):______________________________ |

**Comments:____________________________________________________________________________**

**______________________________________________________________________________________**

**______________________________________________________________________________________**

**______________________________________________________________________________________**

| **Question**  **4.0 RESOURCES** | **Answer** |
| --- | --- |
| **4.1**  **Sources of External Material Support:**  **(tick all that apply)** | ___Family/Self  ___Government  ___Religious institution  ___Other non-governmental organization  ___Individual sponsors/donors/well-wishers  ___No external support  ___Other (specify):______________________________ |
| **4.2**  **Other Sources of Income:**  **(tick all that apply)** | ___Operate a school  ___Farming  ___Selling vegetables  ___Selling charcoal  ___Shop owner  ___Casual Labour  ___Livestock Farming  ___informal Employment  ___Other selling (specify)  ___Other (specify):_____________________________ |
| **4.3**  **Amount of land accessed or owned by organization:** | **Indicate total amount of land owned and/or leased or borrowed:**  ***Owned:***  ___None  ___<¼ acre  ___¼- ½ acre  ___½ -1 acre  ___>1acre: specify_______    ***Leased or Borrowed:***  ___None  ___<¼ acre  ___¼- ½ acre  ___½ -1 acre  ___>1acre: specify_______  **Indicate total amount of land used or accessed for cultivation and/or grazing:**  ***Cultivated:***  ___None  ___<¼ acre  ___¼- ½ acre  ___½ -1 acre  ___>1acre: specify_______  ***Grazing:***  ___None  ___<¼ acre  ___¼- ½ acre  ___½ -1 acre  ___>1acre: specify_______ |
| **4.4**  **Food crops grown by the organization:**  **(tick all crops that apply)** | ___Maize/wheat/other cereals  ___Legumes/beans  ___Roots/Tubers/Potatoes  ___Fruits  ___Vegetables  ___Other (specify): _______________________________  ___None  **If growing crops...**  **Are these crops the organization’s primary food source?**  ___Yes  ___ No  ___ Unsure  ___ Refused to answer  **Are these crops used to *supplement* the organization’s income?**  ___Yes  ___ No  ___ Unsure  ___ Refused to answer |
| **4.5**  **Cash crops grown by the organization:**  **(grown exclusively for income generation)** | ___Tea  ___Coffee  ___Pyrethrum  ___Sugarcane  ___Food crops  ___Other (specify):_______________________________  ___None |
| **4.6**  **How many animals or livestock owned by the organization? (indicate # or tick *none*)** | Cows ___  Goats ___  Sheep ___  Chickens___  Other (specify): __________________________________  ___None  **If animals or livestock owned...**  **Are these animals or livestock used as the organization’s primary food source?**  ___Yes  ___ No  ___ Unsure  ___ Refused to answer  **Are these animals or livestock used to *supplement* the organization’s income?**  ___Yes  ___ No  ___ Unsure  ___ Refused to answer |
| **4.7**  **How many kilometres is the organization from the nearest tarmac road?** | By walking path: ______  By dirt road:_____ |

**Comments:** ______________________________________________________________________________________________________________________________________________________________________________________

___________________________________________________________________________________________

___________________________________________________________________________________________

**5.0 SHELTER CHARACTERISTICS**

| **Question** | **Institutions** |
| --- | --- |
| **5.1**  **Type of building:** | ___No building **If No building, skip to 5.4**  ___Temporary (mud, thatch, etc)  ___Semi-permanent (wood, sheet metal)  ___Permanent (concrete, brick)  ___Other (specify): __________________________ |
| **5.2**  **Does the building have electricity from any source?** | ___Yes in the whole building  ___Yes in some rooms  ___No |
| **5.3**  **What is the roof made of?** | ___Thatch  ___Sheet Metal  ___Wood  ___Shingle  ___No roof |
| **5.4**  **Where does the drinking water come from?** | ___ River, stream, pond, lake, ditch, spring, dam, water vendor  ___ Well, borehole  ___ Public Standpipe (tap in the market, on the plot, or in the village)  ___ Water piped into the home  ___ Purchase bottle water (mineral water) |
| **5.5**  **Toilet facilities:** | ___ Pit latrine How many? ____  ___ Indoor flush toilet How many? ____  ___Other How many? ____  ___None |

**Comments:** ______________________________________________________________________________________________________________________________________________________________________________________

___________________________________________________________________________________________

___________________________________________________________________________________________

**6.0 DIRECTOR CARACTERISTICS**

| **Question** | **Answer** |
| --- | --- |
| **6.1**  **What is the Director’s age?** | Age in years: ____ Year of birth:__________ |
| **6.2**  **What is the Director’s gender?** | ___ Male  ___ Female |
| **6.3**  **What is the Director’s highest level of education?** | ___ None  ___ Primary  ___ Secondary  ___ Vocational  ___ College (specify diploma):________________________  ___ University (specify degree): _______________________  ___ Other (specify): _________________________________ |
| **6.4**  **Is the guardian of children in the program the *legal guardian*?** | ___ Yes for some children  ___ Yes for all children  ___ No  ___ Don’t know  ___ Refuse to answer  ___ Not applicable – no children in residence |
| **6.5**  **Is the Director?** | ___ A well-wisher/volunteer/good Samaritan  ___ A religious missionary  ___ A secular missionary  ___ An employee of a religious or non-governmental organization  ___ An employee of a government organization  ___ Other (specify): _________________________________ |
| **6.6**  **Is the Director Kenyan?** | ___Yes  ___No |
| **6.7**  **Is this the Director’s *primary* job?** | ___ Yes  ___ No  ___ Don’t know  ___ Refuse to answer |

**Comments:** ______________________________________________________________________________________________________________________________________________________________________________________

___________________________________________________________________________________________

___________________________________________________________________________________________

**7.0 INFRASTRUCTURE**

| **Question** | **Answer** |
| --- | --- |
| **7.1**  **How many buildings in the compound (excluding latrines)?** | ___ 1  ___ 2-5  ___ >5  ___ Not applicable, no buildings (**If No buildings,** **Skip to 7.3)** |
| **7.2**  **Is it/are they square, rectangular, or round?** | ___ Square  ___ Rectangular  ___ Round  ___ Other shape  **Please draw out the compound on the back of this paper.** |
| **7.3**  **Number of adults working full-time in facility? (indicate #s)** | Male:____  Female:____ |
| **7.4**  **How many caregivers/staff are present? (indicate #s)** | Day:_____  Night: _____ |
| **7.5**  **How many caregivers/staff are volunteers/paid employees?**  **(indicate #s)** | Paid employees:____  Volunteers:____ |
| **7.6**  **Do you require caretakers/staff/volunteers to have any qualifications?** | ___Yes  ___ No  ___ Unsure  ___ Refused to answer |
| **7.7**  **What forms of transportation are typically used by the organization?** | ___Private vehicles  ___Bicycles  ___Matatu/Bus (Public)  ___Motorbikes (Public)  ___None (walking) |
| **7.8**  **Does the organization own any vehicles?**  **(tick all that apply)** | ___Private car(s)  ___Private bus  ___Motorbikes  ___None |

**Comments:** ______________________________________________________________________________________________________________________________________________________________________________________

___________________________________________________________________________________________

___________________________________________________________________________________________

**8.0 FOOD AND MEALS**

| **Question** | **Answer** |
| --- | --- |
| **8.1**  **Does the organization have a feeding program?** | ___ Yes  ___ No (**If NO, skip to SECTION 9.0**) |
| **8.2**  **How much money does the organization spend on food per week?** | Money spent on food per week: _________  Can you estimate what percent of ***weekly income*** is spent on ***food?*** (ex. 15%, 20%, 45% per week)  ___%  ___unknown |
| **8.3**  **How many meals are eaten a day on site (on average)?** |  |
| **8.3**  **Does everyone eat together?** | ___Yes  ___No  *If no, who eats first? _____________________________*  *If no, who eats last? _____________________________* |
| **8.4**  **What do children typically eat for breakfast? (tick all that apply)** | ___Tea  ___Bread  ___Uji (porridge)  ___Eggs or meat  ___Other (specify) ___________________  ___Nothing/no breakfast served |
| **8.5**  **What do children typically eat for the main meal of the day? (tick all that apply)** | ___Tea  ___Ugali  ___Sukuma Wiki (greens)  ___Cabbage  ___Beans (legumes)  ___Meat or fish  ___Other (specify) __________________________ |

**Comments:** ______________________________________________________________________________________________________________________________________________________________________________________

___________________________________________________________________________________________

___________________________________________________________________________________________

**9.0 SERVICES PROVIDED**

| **Question** | **Answer** |
| --- | --- |
| **9.1**  **Does the organization provide immediate material or financial assistance to children or their families?** | ___Yes to children only  ___Yes to children and families  ___No |
| **9.2**  **What does this assistance consist of? (tick all that apply)** | ___Money  ___School fees  ___School uniforms or other school needs  ___Mattresses and/or blankets  ___Household repairs  ___Bed-nets  ___Transportation  ___Food items  ___Seeds or agricultural inputs  ___Other (specify):__________________________________ |
| **9.3**  **What other kinds of assistance are provided? (tick all that apply)** | ___ Emotional support  ___ Feeding program  ___ Sanitary pads  ___ Medical assistance  ___ Social work  ___ Informal education / vocational training  ___ Day-care for pre-school aged children  ___ Transportation services  ___ Emergency shelter  ___ Long-term shelter  ___ Other (specify):___________________________________ |
| **9.5**  **Leisure: Is there...? (tick all that apply)** | ___ Scheduled leisure time  ___ Space dedicated to leisure activities  ___ Books available on site  ___ Toys available on site  ___ Games available on site  ___ Television available on site  ___ Playground on site or nearby |
| **9.6**  **Sports activities: Are there...? (tick all that apply)** | ___ Space or facilities for any sports existing (e.g. football field)  ___ Sports equipment available on site  ___ Organized sports programs for children  ___ Adult care-takers involved in coaching or organizing sports |
| **9.7**  **What tasks do children assist with at the organization? (tick all that apply)** | ___ Child care  ___ Cooking  ___ Cleaning  ___ Water collection  ___ Firewood collection  ___ Food gathering  ___ Income generating activities (selling wares, begging, etc.)  ___ Animal care (including herding)  ___Other (specify): _______________________________  ___None |
| **9.8**  **Emotional and psychosocial support: Is there....? (tick all that apply)** | ___ Individual counselling  ___ Support groups  ___ Drug rehabilitation  ___ Nothing formal  ___ Informal one on one time with adults  ___ Other (specify): _______________________________ |
| **9.9**  **Religious education and experiences** | **Do participants attend any of the following at your organization**:  ___ Church Services  ___ ‘Sunday School’  ___ Other religious education  ***Is it:***  ___ Daily  ___ Weekly  ___ Other (specify): _______________________________  ___ Not applicable  Is it:  ___Compulsory  ___Voluntary |
| **9.10**  **Where is the child first taken when sick?** | ___Traditional healer  ___Spiritual healer or religious leader  ___Public clinic or hospital  ___Private clinic or hospital  ___ On-site healthcare worker  ___Other (specify): ________________________________ |
| **9.11**  **Life skills training provided by organization? (tick all that apply)** | ___Cooking  ___Money management  ___Business development  ___Time management  ___Trades  ___Communication skills  ___Psychosocial skills  ___ Other (specify): _______________________________ |

**Comments:** ______________________________________________________________________________________________________________________________________________________________________________________

___________________________________________________________________________________________

___________________________________________________________________________________________

**10.0 POLICIES**

| **Question** | **Answer** |
| --- | --- |
| **10.1**  **What are the admission criteria for the organization? (tick all that apply)** | ___Family member or child of friend  ___Age (specify in 10.2)  ___HIV-positive  ___HIV-negative  ___Any orphan  ___Double orphan  ___On or of the street child/youth  ___Abused or abandoned  ___Any child in need  ___Other (specify): ________________________ |
| **10.2a**  **Is there a lower age limit?** | ___Yes: ________Years  ___No |
| **10.2b**  **Is there an upper age limit?** | ___Yes: ________Years  ___No |
| **10.3**  **What are the criteria for expulsion?** | ___Violence  ___Drug use  ___Disobedience  ___Abuse of other children  ___Other (specify):___________________________  ___No expulsion policy |
| **10.4**  **How is discipline enforced?** | ___Child psychology  ___Scolding  ___Corporal punishment  ___Isolation of child  ___Withholding of food or other material needs  ___Withholding of privileges  ___ More chores  ___Other (specify): _______________________________ |
| **10.5**  **If corporal punishment is used:** | ___ Corporal punishment not used **– skip to 10.6**  *Who does it?* ____ Head of household/Director  ____ Immediate caregiver  ____ Other: _______________________  *With what is it applied?*  ___Hand  ___Closed fist  ___Belt  ___Paddle  ___Stick or other object  ___Other (specify): ___________________  *Where on the child’s body is it applied?*  ___ Buttocks  ___ Hands  ___ Back  ___ Face/Head  ___Anywhere within reach  ___ Other: (specify) _____________________________ |
| **10.6**  **What types of health promotion are offered by the organization? (tick all that apply)** | ___Hygiene and sanitation education  ___Immunization program  ___Nutrition program  ___TB program  ___HIV program  ___No health promotion programs |
| **10.7**  **What information or tools for HIV prevention are provided? (tick all that apply)** | ___Education  ___Condom availability  ___HIV counselling and testing  ___None  ___Refuse to answer |

**Comments:** ______________________________________________________________________________________________________________________________________________________________________________________

___________________________________________________________________________________________

___________________________________________________________________________________________

**11.0 FAMILY LINKAGES**

| **Question** | **Answer** |
| --- | --- |
| **11.1**  **Does the organization have a policy or program on family integration or family connections?** | ___ Yes  ___ No  ___ Not applicable  ___Refuse to answer  **If no, skip to 11.3** |
| **11.2**  **What does this consist of?** | ___ Attempted repatriation  ___ Regular contact with the family  ___ Ensuring children know who their parents are/were  ___ Family support programs  ___ Not applicable (no policy or program)  ___ Refuse to answer |
| **11.3**  **Are the children allowed to know about their parentage, both who they are/were and how they came to be living without them?** | ___Yes  ___No  ___ Not applicable  *If yes, at what age is the issue introduced?*  ______ |
| **11.4**  **Do children typically know their parental/family history?** | ___ Yes  ___ No  ___ Unsure  ___Refuse to answer |
| **11.5**  **How many children have a birth certificate?**  **(of children in residence)** |  |
| **11.6**  **Do children have another form of identification?** | ___ Yes (specify):___________________________  ___ No  ___ Unsure  ___Refuse to answer |

**Comments:** ______________________________________________________________________________________________________________________________________________________________________________________

______________________________________________________________________________________________________________________________________________________________________________________

**12.0 ORGANIZATION FOOD SECURITY**

***Please consider what happened in the last 30 days (1 month):***

***For each of the following questions, please answer whether this happened never, rarely (once or twice), sometimes (3-10 times), or often (more than 10 times) in the last month.***

| - 1. **Did you worry that your organization would not have enough food?**   □Never (0) □Rarely (1) □Sometimes (2) □Often (3) □Not applicable (4) |
| --- |
| - 1. **Was any organization member not able to eat the kinds of foods they preferred because of a lack of resources?**   □Never (0) □Rarely (1) □Sometimes (2) □Often (3) □Not applicable (4) |
| - 1. **Did any organization member eat just a few kinds of food day after day due to a lack of resources?**   □Never (0) □Rarely (1) □Sometimes (2) □Often (3) □Not applicable (4) |
| - 1. **Did any organization member eat food that they preferred not to eat because of a lack of resources to obtain other types of food?**   □Never (0) □Rarely (1) □Sometimes (2) □Often (3) □Not applicable (4) |
| - 1. **Did any organization member eat a smaller meal than you felt the child needed because there was not enough food?**   □Never (0) □Rarely (1) □Sometimes (2) □Often (3) □Not applicable (4) |
| - 1. **Did any other organization member eat fewer meals in a day because there was not enough food?**   □Never (0) □Rarely (1) □Sometimes (2) □Often (3) □Not applicable (4) |
| - 1. **Was there ever no food at all in your organization because there were not resources to get more? (Were your organization food stores ever completely empty and there was no way of getting more?)**   □Never (0) □Rarely (1) □Sometimes (2) □Often (3) □Not applicable (4) |
| - 1. **Did any organization member go to sleep at night hungry because there was not enough food?**   □Never (0) □Rarely (1) □Sometimes (2) □Often (3) □Not applicable (4) |
| - 1. **Did any organization member go a whole day without eating anything because there was not enough food?**   □Never (0) □Rarely (1) □Sometimes (2) □Often (3) □Not applicable (4) |

**Comments:** ______________________________________________________________________________________________________________________________________________________________________________________

____________________________________________________________________________________________________________________________________________________________________________­­­­­­­­­
