# Supplementary material for: Models of care for orphaned and separated children and upholding children’s rights: cross-sectional evidence from western Kenya
Source: BMC Int Health Hum Rights. 2014 Apr 1;14:9. doi: 10.1186/1472-698X-14-9 (PMC4021203; doi:10.1186/1472-698X-14-9)
Supplement: Additional file 2 — Site Assessment (Households and Institutions). [file 1472-698X-14-9-S2.doc]

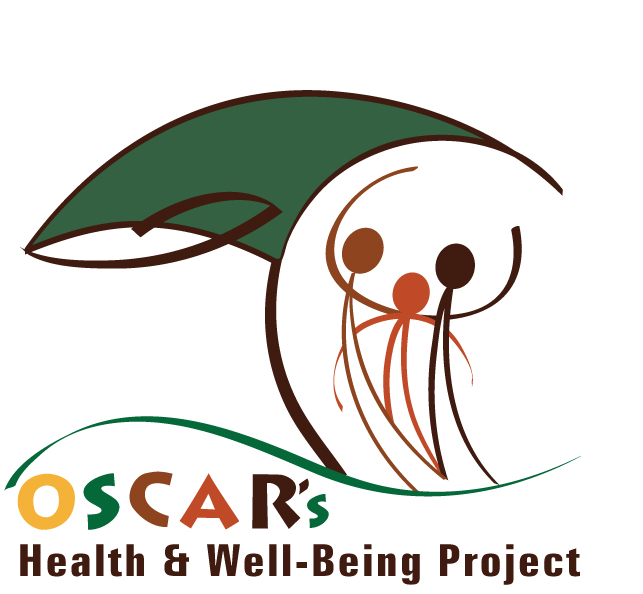


***Site Assessment (Households and Institutions)***

**Household/Facility Code: _________________**

**Date: ________________________________**

**Person administering questionnaire: _______________________________________________**

***These questions are to be asked of the Head of the Household or Director or In-Charge.***

***1.0 Contact Information:***

| **1.1**  **Name of Household Head, Director or Guardian (specify role):** |  |
| --- | --- |
| **1.2**  **Name of Facility:** |  |
| **1.3**  **Location, Sub-Location, Village:** |  |
| **1.4**  **Contact phone numbers:** |  |
| **1.5**  **Email address and or website:** |  |
| **1.6**  **GPS Coordinates:** |  |

***2.0 Shelter – General***

| **Question** | **Answer** |
| --- | --- |
| **2.1**  **Type of Environment** | ___Registered Charitable Children’s Institution (CCI)  ___ Non-Governmental Orphanage (i.e. long-term care)  ___ Government Orphanage (i.e. long-term care)  ___ Non-Governmental Foster home (short-term care)  ___ Private  ___Private family home  ___Unregistered Orphanage (i.e. long-term care)  ___Unregistered Foster Home (i.e. short-term care)  ___Boarding school  ___ Statutory  ___ Temporary Shelter  ___ Rehabilitation Centre  ___ Correctional Centre/Remand  ___Other (specify): ______________________________ |
| **2.2**  **When did the residence begin accommodating orphaned children?** |  |
| **2.3**  **Governance Structure** | ___Village Chief or Elders  ___ Head of household  ___Private individual  ___Single Remunerated In-Charge/ Director (no Board)  ___Single Volunteer In-Charge/ Director (no Board)  ___Remunerated Board of Directors/Trustees  ___Volunteer Board of Directors/Trustees  ___Other Advisory Committee  ___Other (specify): _________________________________ |

**Comments:** ______________________________________________________________________________________________________________________________________________________________________________________

**3.0 Children in Residence**

| **Question** | **Answer** |
| --- | --- |
| **3.1**  **What is the maximum capacity of the home (the maximum number of children that are supposed to be care for)?** | Day: _____  Night: ____ |
| **3.2**  **What are the age criteria (if any) for accepting children?** |  |
| **3.3**  **How many children are there in the residence?** | Aged 0-5 Boys: Girls:  Aged 6-10 Boys: Girls:  Aged 10-17 Boys: Girls:  Aged 17+ Boys: Girls: |
| **3.4**  **How many children are there because either or both their parents are deceased or absent from their lives?** | Aged 0-5 Boys: Girls:  Aged 6-10 Boys: Girls:  Aged 10-17 Boys: Girls:  Aged 17+ Boys: Girls: |

**Comments:** ______________________________________________________________________________________________________________________________________________________________________________________

**4.0 Resources**

| **Question** | **Answer** |
| --- | --- |
| **4.1**  **Sources of External Material Support:** | ___Family  ___Government  ___Religious institution  ___Other non-governmental organization  ___Individual sponsors/donors/well-wishers  ___Other  ___No external support |
| **4.2**  **Other Sources of Income:** | ___Operate a school  ___Farming  ___Selling vegetables  ___Selling charcoal  ___Shop owner  ___Casual Labour  ___Livestock Farming  ___Formal Employment  ___Begging  ___Commercial Sex Work  ___Other selling (specify)  ___Other |
| **4.3**  **Amount of land accessed or owned by household or facility:** | ***Owned:***  ___None  ___<¼ acre  ___¼- ½ acre  ___½ -1 acre  ___>1acre: specify_______    ***Cultivated:***  ___None  ___<¼ acre  ___¼- ½ acre  ___½ -1 acre  ___>1acre: specify_______  ***Leased or Borrowed:***  ___None  ___<¼ acre  ___¼- ½ acre  ___½ -1 acre  ___>1acre: specify_______  ***Grazing:***  ___None  ___<¼ acre  ___¼- ½ acre  ___½ -1 acre  ___>1acre: specify_______ |
| **4.4**  **Food crops grown by the household or facility:** | ___Maize/wheat/other cereals  ___Legumes/beans  ___Roots/Tubers/Potatoes  ___Fruits  ___Vegetables  ___Other:  ___None |
| **4.5**  **Cash crops grown by the household or facility:** | ___Tea  ___Coffee  ___Pyrethrum  ___Sugarcane  ___Food crops  ___Other:  ___None |
| **4.6**  **Animals or livestock (number) owned by the household or facility:** | Cows ___  Goats ___  Sheep ___  Chickens___  Other: |
| **4.7**  **How many kilometres is the home from the nearest tarmac road?** |  |
| **4.8**  **How is the tarmac road accessed?** | ___Foot path  ___Dirt road  ___Both |
| **4.9**  **Transportation available to household or facility** | ___Foot  ___Bicycle  ___Public Transport  ___Private vehicle |

**Comments:** ______________________________________________________________________________________________________________________________________________________________________________________

**5.0 Shelter Characteristics**

| **Question** | **Institutions** |
| --- | --- |
| **5.1**  **Type of shelter (for sleeping):** | ___Temporary (mud, thatch, etc)  ___Semi-permanent (wood, sheet metal)  ___Permanent (concrete, brick)  ___Other |
| **5.2**  **Does the shelter have electricity from any source?** | ___Yes in the whole building  ___Yes in some rooms  ___No |
| **5.3**  **What is the roof made of?** | ___Thatch  ___Sheet Metal  ___Wood  ___Shingle  ___No roof |
| **5.4**  **Where does the drinking water come from?** | ___ River, stream, pond, lake, ditch, spring, dam, water vendor  ___ Well, borehole  ___ Public Standpipe (tap in the market, on the plot, or in the village)  ___ Water piped into the home  ___ Purchase bottle water (mineral water) |
| **5.5**  **Toilet facilities:** | ___ Pit latrine How many? ____  ___ Indoor flush toilet How many? ____  ___Other How many? ____ |

**Comments:** ______________________________________________________________________________________________________________________________________________________________________________________

**6.0 Guardian Characteristics**

| **Question** | **Answer** |
| --- | --- |
| **6.1**  **What is the guardian’s age?** | Age in years: ____ Year of birth:__________  ___Child has no guardian |
| **6.2**  **Is the guardian legally mandated?** | ___ Yes  ___ No  ___ Don’t know  ___ Refuse to answer |
| **6.3**  **In relation to the child or children, is the legal guardian** | ___ A family member/relative (specify below):  ___Mother  ___Father  ___Sibling  ___Grandmother  ___Grandfather  ___Paternal auntie  ___Paternal uncle  ___Maternal auntie  ___Maternal uncle  ___ Other_______________  ___ A friend  ___ A religious missionary  ___ A secular missionary  ___ An employee of a religious or non-governmental organization  ___ An employee of a government organization  ___ Other |
| **6.4**  **What is the guardian’s highest level of education?** | ___ None  ___ Primary  ___ Secondary  ___ Vocational  ___ University  ___ Other |
| **6.5**  **Is the guardian Kenyan?** | ___Yes  ___No |
| **6.6**  **Does the guardian live in the same place as the child?** | ___ Same compound, different house  ___ Same house  ___ Somewhere else  ___ Legal guardian deceased or absent |

**Comments:** ______________________________________________________________________________________________________________________________________________________________________________________

**7.0 Living and Sleeping Arrangements**

| **Question** | **Answer** |
| --- | --- |
| **7.1**  **How many buildings in the compound (excluding latrines)?** | ___ 1  ___ 2-5  ___ >5 |
| **7.2**  **Is it/are they square, rectangular, or round?** | ___ Square  ___ Rectangular  ___ Round  ___ Other shape  **Please draw out the compound on the back of this paper.** |
| **7.3**  **Number of adults living full-time in household or facility?** |  |
| **7.4**  **How many caregivers are present:** | Day:_____  Night: _____ |
| **7.5**  **Do boys and girls sleep separately?** | ___Yes  ___No |
| **7.6**  **Are children of similar ages clustered together in rooms, beds, or on mattresses?** | ___Yes  ___No |
| **7.7**  **How many beds/mattresses in total?** |  |
| **7.8**  **Are siblings or children from same village housed together?** | ___Yes  ___No |
| **7.9**  **Is there some kind of cushion on each occupied bed?** | ___Foam  ___Store bought mattress  ___Hay or straw  ___Other  ___None |
| **7.10**  **Does each child have his/her own bed/mattress?** | ___Always  ___Sometimes  ___No |
| **7.11**  **How many blankets does each child have?** | ___Not all children have at least one blanket  ___One  ___Two  ___More than Two |
| **7.12 Does each bed/mattress have a bed net?** | ___Yes  ___No |
| **7.13 Does each child have his/her own private cabinet, metal box, trunk, or drawer?** | ___Yes  ___No |

**Comments:** ______________________________________________________________________________________________________________________________________________________________________________________

**8.0 Food and Meals**

| **Question** | **Answer** |
| --- | --- |
| **8.1**  **Proportion of household income spent on food** |  |
| **8.2**  **How many meals are eaten a day (on average)?** |  |
| **8.3**  **Does everyone eat together?** | ___Yes  ___No  *If no, who eats first? _____________________________*  *If no, who eats last? _____________________________* |
| **8.4**  **What do children typically eat for breakfast? (tick all that apply)** | ___Tea  ___Bread  ___Uji (porridge)  ___Eggs or meat  ___Other (specify) ___________________  ___Nothing |
| **8.5**  **What do children typically eat for the main meal of the day? (tick all that apply)** | ___Tea  ___Ugali  ___Sukuma Wiki (greens)  ___Cabbage  ___Beans (legumes)  ___Meat or fish  ___Other (specify) __________________________ |

**Comments:** ______________________________________________________________________________________________________________________________________________________________________________________

**9.0 Material, Emotional, and Psychological Needs**

| **Question** | **Answer** |
| --- | --- |
| **9.1**  **Does each child have at least one pair of shoes?** | ___Yes  ___No |
| **9.2**  **Does each child have 2 pairs of (not school uniforms) clothing?** | ___Yes  ___No |
| **9.3**  **How many uniforms does each school-going child have?** | ___1  ___>1  ___Not all children have a complete uniform |
| **9.4**  **How many children are in school?** | ___ Aged 5-13  ___ Aged 14-17  ___ Aged 17+ |
| **9.5**  **Leisure: Is there...?** | ___ Scheduled leisure time  ___ Space dedicated to leisure activities  ___ Books available on site  ___ Toys available on site  ___ Games available on site  ___ Television available on site  ___ Playground on site or nearby |
| **9.6**  **Sports activities: Are there...?** | ___ Space or facilities for any sports existing (e.g. football field)  ___ Sports equipment available on site  ___ Organized sports programs for children  ___ Adult care-takers involved in coaching or organizing |
| **9.7**  **What household tasks do children assist with?** | ___ Child care  ___ Cooking  ___ Water collection  ___ Firewood collection  ___ Food gathering  ___ Income generating activities (selling wares, begging, etc.)  ___ Animal care (including herding)  ___Other  ___None |
| **9.8**  **Emotional and psychosocial support** | ___ Individual counselling  ___ Support groups  ___ Drug rehabilitation  ___ Nothing formal  ___ Informal one on one time with adults  ___ Other |
| **9.9**  **Religious education** | ___ Compulsory religious education  ___ Voluntary religious education  *Is it:*  ___ Daily  ___ Weekly  ___ Other  ___ Not applicable |
| **9.10**  **Where is the child first taken when sick?** | ___Traditional healer  ___Spiritual healer or religious leader  ___Public clinic or hospital  ___Private clinic or hospital  ___Other (specify): ________________________________ |
| **9.11**  **Are the children medically insured?** | ___Yes  ___No  ___Don’t know  ___Refuse to answer |
| **9.12**  **Life skills training provided by household or facility?** | ___Cooking  ___Money management  ___Business development  ___Time management  ___Trades  ___Communication skills |

**Comments:** ______________________________________________________________________________________________________________________________________________________________________________________

**10.0 Policies**

| **Question** | **Answer** |
| --- | --- |
| **10.1**  **What are the admission criteria for the shelter or home?** | ___Family member or child of friend  ___Age (specify)  ___HIV-positive  ___HIV-negative  ___Any orphan  ___Double orphan  ___On or of the street  ___Abused or abandoned  ___Other (specify): ________________________ |
| **10.2**  **Is there an upper age limit for residence?** | ___Yes: ________Years  ___No |
| **10.3**  **What are the criteria for explusion?** | ___Violence  ___Drug use  ___Disobedience  ___ Abuse of other children  ___Other |
| **10.4**  **How is discipline enforced?** | ___Child psychology  ___Scolding  ___Corporal punishment  ___Isolation of child  ___Withholding of food or other material needs  ___Withholding of privileges  ___ More chores  ___Other |
| **10.5**  **If corporal punishment is used:** | ___ Corporal punishment not used – skip to 10.6  *Who does it?* ____ Head of household/Director  ____ Immediate caregiver  ____ Other: _______________________  *With what is it applied?*  ___Hand  ___Closed fist  ___Belt  ___Paddle  ___Stick or other object  ___Other: ___________________  *Where on the child’s body is it applied?*  ___ Buttocks  ___ Hands  ___ Back  ___ Face/Head  ___Anywhere within reach  ___ Other: (specify) _____________________________ |
| **10.6**  **What information or tools for HIV prevention are provided?** | ___Education  ___Condom availability  ___HIV testing  ___None  ___Refuse to answer |

**Comments:** ______________________________________________________________________________________________________________________________________________________________________________________

**11.0 Family linkages**

| **Question** | **Answer** |
| --- | --- |
| **11.1**  **Do they have a policy or program on family integration or family connections?** | ___ Yes  ___ No  ___ Not applicable  ___Refuse to answer |
| **11.2**  **What does this consist of?** | ___ Attempted repatriation  ___ Regular contact with the family  ___ Ensuring children know who their parents are/were  ___ Family support programs  ___ Not applicable (no policy or program)  ___ Refuse to answer |
| **11.3**  **Are the children allowed to know about their parentage, both who they are/were and how the came to be living without them?** | ___Yes  ___No  *If yes, at what age is the issue introduced?*  ______ |
| **11.4**  **Do children typically know their parental/family history?** | ___ Yes  ___ No  ___ Not applicable  ___Refuse to answer |
| **11.5**  **How many children have a birth certificate?** |  |

**Comments:** ______________________________________________________________________________________________________________________________________________________________________________________

**12.0 Household Food Security**

***Please consider what happened in the last 30 days (1 month):***

***For each of the following questions, please answer whether this happened never, rarely (once or twice), sometimes (3-10 times), or often (more than 10 times) in the last month.***

| - 1. **Did you worry that your household would not have enough food?**   □Never (0) □Rarely (1) □Sometimes (2) □Often (3) |
| --- |
| - 1. **Was any household member not able to eat the kinds of foods they preferred because of a lack of resources?**   □Never (0) □Rarely (1) □Sometimes (2) □Often (3) |
| - 1. **Did any household member eat just a few kinds of food day after day due to a lack of resources?**   □Never (0) □Rarely (1) □Sometimes (2) □Often (3) |
| - 1. **Did any household member eat food that they preferred not to eat because of a lack of resources to obtain other types of food?**   □Never (0) □Rarely (1) □Sometimes (2) □Often (3) |
| - 1. **Did any household member eat a smaller meal than you felt the child needed because there was not enough food?**   □Never (0) □Rarely (1) □Sometimes (2) □Often (3) |
| - 1. **Did any other household member eat fewer meals in a day because there was not enough food?**   □Never (0) □Rarely (1) □Sometimes (2) □Often (3) |
| - 1. **Was there ever no food at all in your household because there were not resources to get more? (Were your household food stores ever completely empty and there was no way of getting more?)**   □Never (0) □Rarely (1) □Sometimes (2) □Often (3) |
| - 1. **Did any household member go to sleep at night hungry because there was not enough food?**   □Never (0) □Rarely (1) □Sometimes (2) □Often (3) |
| - 1. **Did any household member go a whole day without eating anything because there was not enough food?**   □Never (0) □Rarely (1) □Sometimes (2) □Often (3) |

**Comments:** ______________________________________________________________________________________________________________________________________________________________________________________
